# Marked improvement in leptomeningeal carcinomatosis and spinal cord metastases following alectinib treatment of crizotinib-resistant, ALK-positive lung adenocarcinoma

**DOI:** 10.1007/s13691-015-0231-9

**Published:** 2015-07-18

**Authors:** Hidehiko Kuribayashi, Shinji Abe, Naoyuki Kuse, Yuji Kusunoki, Ritsuko Narato, Hitoshi Saito, Akihiko Gemma

**Affiliations:** 1 Department of Pulmonary Medicine, Tokyo Metropolitan Hiroo General Hospital, 2-34-10 Ebisu Shibuya-ku, Tokyo, 150-0013 Japan; 2 Department of Pulmonary Medicine and Oncology, Graduate School of Medicine, Nippon Medical School, 1-1-5 Sendagi Bunkyo-ku, Tokyo, 113-8602 Japan

**Keywords:** Anaplastc lymphoma kinase (ALK)-positive non-small cell lung cancer (NSCLC), Alectinib, Spinal cord metastases, Leptomeningeal carcinomatosis

## Abstract

We report a case of 50-year-old Japanese female with anaplastic lymphoma kinase (ALK)-positive, crizotinib-resistant lung adenocarcinoma, whose leptomeningeal carcinomatosis and spinal cord metastases were dramatically improved by the second-generation ALK inhibitor alectinib. Magnetic resonance imaging (MRI) revealed multiple brain metastases at diagnosis of lung cancer. Carboplatin/paclitaxel/bevacizumab chemotherapy was administered, but enlargement of brain tumors was observed after 3 months. Gamma knife radiosurgery was performed and then the patient received second-line chemotherapy with crizotinib. After 4 months brain MRI revealed the development of leptomeningeal carcinomatosis. Despite the patient undergoing whole brain radiotherapy, spinal cord metastases appeared. Third-line chemotherapy with alectinib was initiated for the management of metastases in central nervous system (CNS) including those in the leptomeninges and spine cord. After 3 months, marked tumor responses were observed in both the leptomeningeal carcinomatosis and spinal cord metastases. This report suggests that alectinib is a promising drug for ALK-positive lung adenocarcinoma with CNS metastases.

## Introduction

Leptomeningeal carcinomatosis and spinal cord metastases have recently emerged as important complications in recurrent, driver mutation-positive non-small cell lung cancer (NSCLC). Although epidermal growth factor receptor tyrosine kinase inhibitors (EGFR-TKIs) are effective for EGFR-positive NSCLC patients with brain metastases, patients treated with EGFR-TKIs over a period of months may have an increased risk of developing central nervous system (CNS) metastases. Lee et al. reported that the CNS is a frequent relapse site, occurring in 13 % of NSCLC patients who experienced benefit with EGFR-TKIs [1]. In patients with anaplastic lymphoma kinase (ALK)-positive NSCLC, the first ALK-TKI crizotinib has significantly improved objective response rates compared with cytotoxic chemotherapy [2]. However, it has been recognized that CNS relapse is common after crizotinib treatment in particular leptomeningeal carcinomatosis and spinal cord metastases, which both have extremely poor prognoses [3–5]. Because radiotherapy is unsuitable for intramedullary and spinal cord metastases, the optimal management of this complication needs to be investigated.

## Case report

**Fig. 1:**
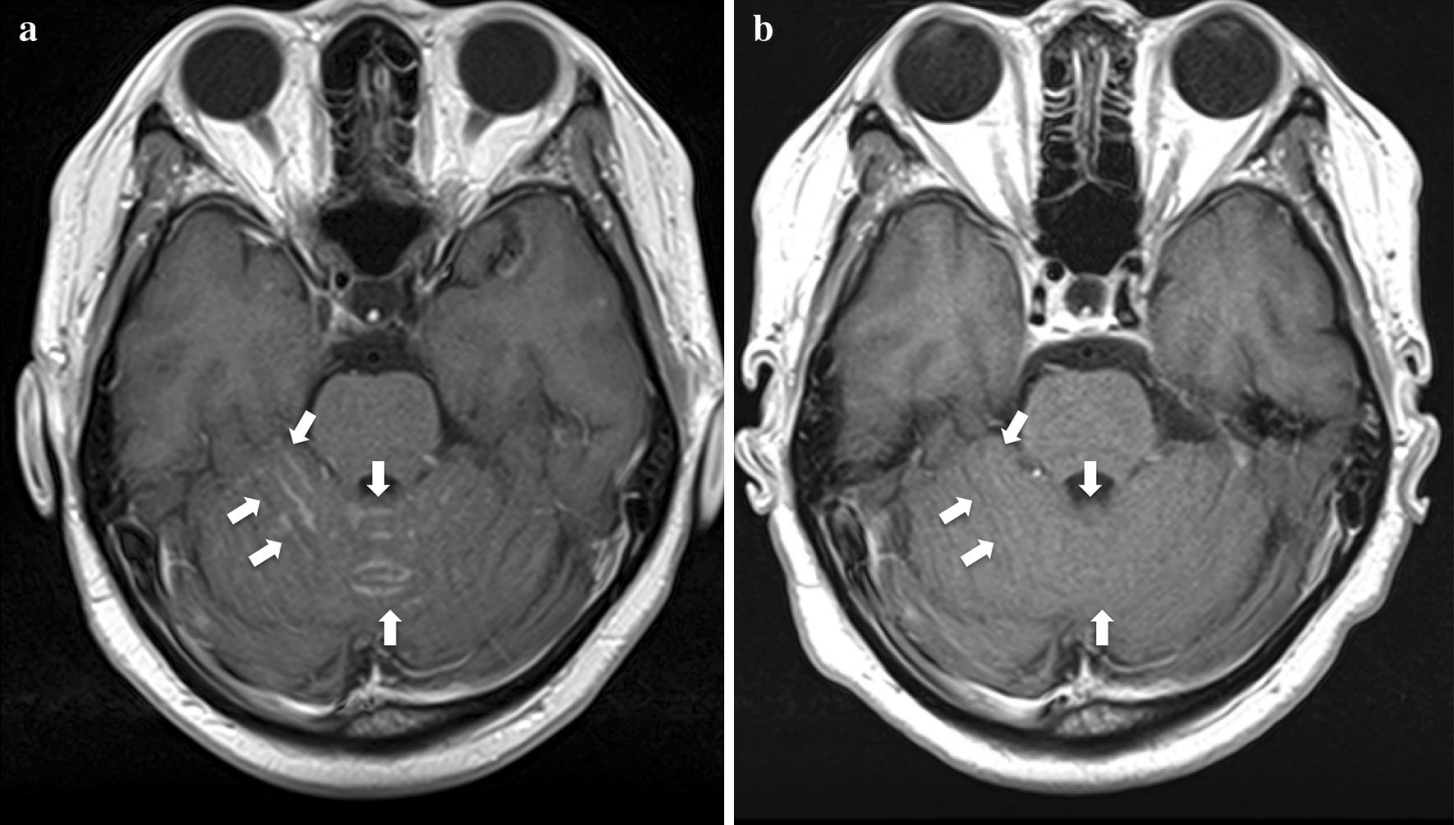
T1-weighted post-gadolinium MRI before **a** and after **b** 3 months of alectinib treatment revealed a marked response in leptomeningeal carcinomatosis (see *arrows*)

**Fig. 2:**
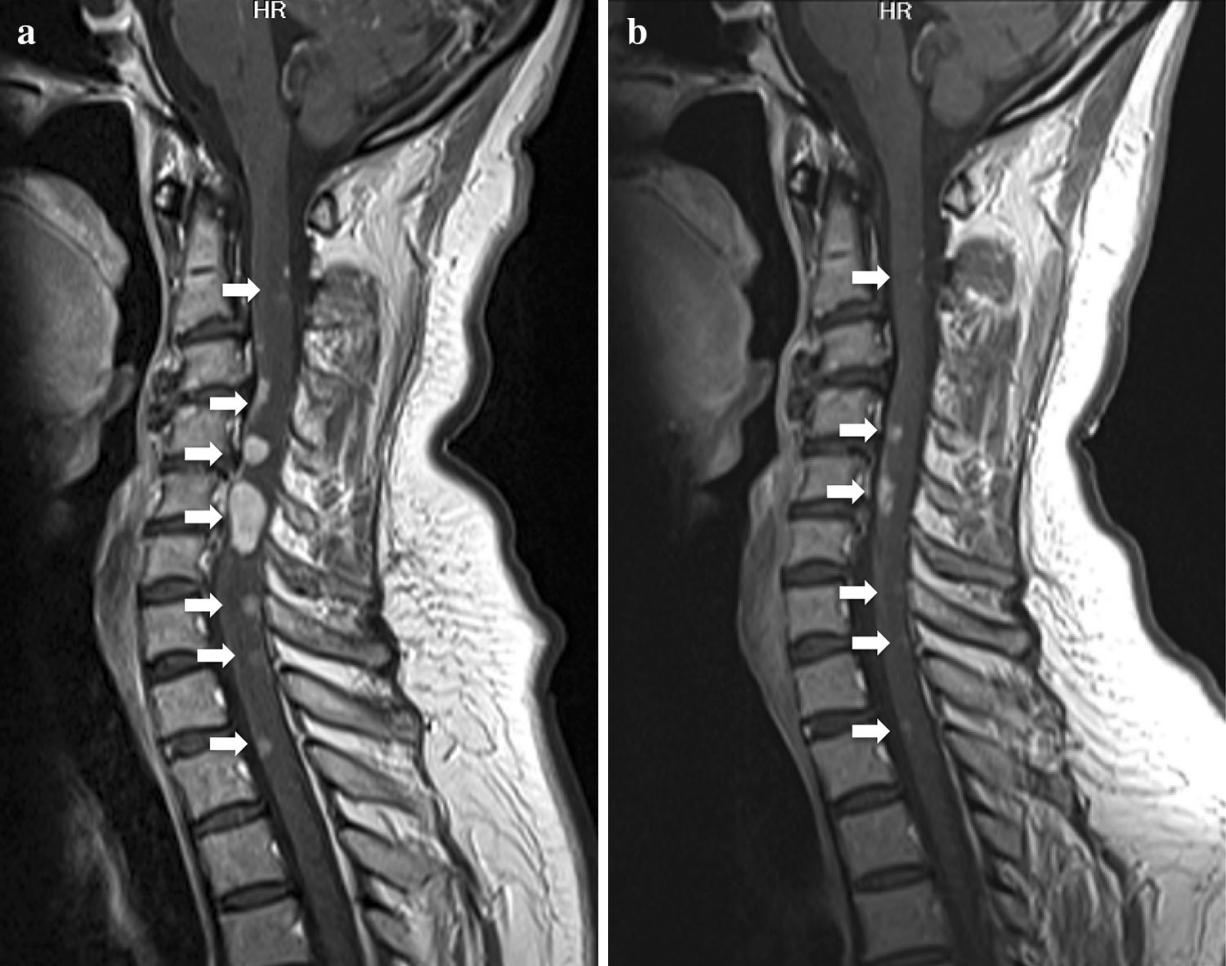
T1-weighted post-gadolinium MRI scans before **a** and after **b** 3 months of alectinib treatment revealed a marked response in multiple spinal cord metastases (see *arrows*)

A 50-year-old female who had never smoked presented with a cough for 4 months and dyspnea for 3 weeks. Chest computed tomography (CT) scans revealed a lung mass in the left upper lobe, multiple nodules in both lungs, and several hilar and mediastinal lymphadenopathy. The patient was diagnosed with poorly differentiated stage IV lung adenocarcinoma (clinical T4N3M1b), which was ALK-positive by immunostaining with ALK antibody. Fluorescence in situ hybridization (FISH) analysis for ALK gene rearrangement was inconclusive. Brain MRI revealed asymptomatic multiple parenchymal metastases. First-line chemotherapy with carboplatin/paclitaxel/bevacizumab was administered, resulting in stable disease after 4 cycles. Despite an improvement in systemic disease, brain MRI after 3 months demonstrated tumor enlargement. The patient underwent gamma knife radiosurgery against multiple brain metastases and second-line chemotherapy with crizotinib was initiated. After 4 months of crizotinib treatment, partial responses were detected in the primary tumor site, and intrapulmonary and lymph node metastases, but brain MRI indicated that leptomeningeal carcinomatosis had developed. The patient received whole brain radiotherapy (WBRT), after which she experienced sharp pains in the arms and neck. T1 brain MRI revealed no change in leptomeningeal carcinomatosis (Fig. 1a), whereas T1 neck MRI led to the detection of new metastases in the cervical spinal cord (Fig. 2a). Because of the wide distribution of spinal cord metastases, radiation therapy was contraindicated. To manage the CNS metastases, including those in the leptomeninges and spinal cord, third-line chemotherapy with alectinib (600 mg/day) was initiated. The patient reported a gradual improvement in arm and neck pain. After 3 months of alectinib treatment, T1 MRI demonstrated a marked reduction in leptomeningeal carcinomatosis (Fig. 1b) and spinal cord metastases (Fig. 2b). Alectinib was well tolerated, with no significant adverse events.

## Discussion

The CNS is an important site of metastases in ALK-positive NSCLC. Among patients previously treated with crizotinib in a clinical trial of the second-generation ALK inhibitor ceritinib, the rate of CNS metastases was almost 60 % [6]. Although crizotinib has led to significant improvements in objective response rates and progression-free survival compared with cytotoxic chemotherapy in patients with ALK-positive NSCLC [2], the antitumor effect of crizotinib in the CNS is limited. A study by Maillet et al. demonstrated that in two patients with ALK-positive NSCLC, crizotinib was effective for extracranial disease, but ineffective against intracranial metastases [3]. Ou et al. reported that CNS metastasis is the leading cause of treatment failure in ALK-positive NSCLC patients treated with crizotinib [4]. The cerebrospinal fluid (CSF)-to-plasma ratio of crizotinib is estimated be only 0.0026, suggesting that the drug has poor blood–brain barrier (BBB) penetration [7].

Because patients with spinal cord metastases are ineligible for radiotherapy, improvements in BBB penetration and the potency of anticancer drug is necessary for patients with ALK-positive NSCLC. It is expected that alectinib will be a key drug in the treatment strategy for CNS metastases in this patient population. A phase I/II trial revealed that alectinib was well tolerated in patients with ALK-rearranged NSCLC who were resistant or intolerant to crizotinib, including those with CNS metastases [8]. In a recent trial by Gainor et al. among 21 crizotinib-resistant ALK-positive NSCLC patients with CNS metastases, 52 % had objective responses in metastatic CNS involvement. Three of four alectinib-treated patients with leptomeningeal carcinoma who were treated with alectinib experienced radiographic and clinical improvements, while one had stable disease according to imaging [9]. Because alectinib is not transported by *P*-glycoprotein which is a key factor in BBB penetration, it may be beneficial for CNS metastases in crizotinib-treated patients [10]. Nevertheless, further research into the pharmacological and clinical properties of ALK inhibitors is needed.

This report covers a patient with ALK-positive, crizotinib-resistant lung adenocarcinoma who experienced a marked improvement in leptomeningeal carcinoma and spinal cord metastases following 3 months of alectnib treatment. To the best of authors’ knowledge, this is the first case report to demonstrate that alectinib was therapeutically effective for cervical spinal cord metastases. This finding suggests that alectinib is a promising drug for ALK-positive NSCLC patients with CNS metastases.
